# Reply to Spellberg et al., “Static vs cidal: it’s not complex; it’s simply incorrect”

**DOI:** 10.1128/aac.00615-25

**Published:** 2025-07-21

**Authors:** Teresa Gil-Gil, Brandon A. Berryhill

**Affiliations:** 1 Department of Biology, Emory University 123382, Atlanta, Georgia, USA; Columbia University Irving Medical Center, New York, New York, USA

## REPLY

We are flattered by this diverse collective’s interest (1) in our article, and we agree with this collective scientifically but have disagreements both linguistically and strategically. As we demonstrate in Fig, 1, the concentrations where traditional bacteriostatic agents appear to simply inhibit growth are extremely narrow (2). These drugs more generally either actually kill or fail to control the treated bacteria. Indeed, we have previously shown that even at the concentration where the drug appears truly bacteriostatic, there are no cells in bacteriostasis; rather, the rate of population growth is equal to the rate of death (3). Scientifically, we agree that there is no meaningful difference between the drugs that are traditionally considered bacteriostatic and those considered bactericidal.

Our first disagreement with the collective is one of linguistics, primarily their qualm with our first sentence. To state this sentence another way, the outcome of treatment with these agents is more complex than the description of the agent as bacteriostatic or bactericidal alone would imply. Rifampin is canonically considered bacteriostatic for *Escherichia coli,* while it is bactericidal for Mycobacterium (4); here, the context-dependence for the nature of the drug is the underlying pathogen being treated. The bacteriostatic or bactericidal nature of chloramphenicol is determined by the treated cell’s ability to produce (p)ppGpp (5); here, the complexity is in the physiological state of the bacterium. However, in spite of these microbiological discrepancies, multiple meta-analyses have found little difference in patient outcomes between traditionally bacteriostatic and bactericidal drugs, as is well recognized (5, 6). We will concede that the clarity of this sentence is lower than we would have liked. Perhaps we should have mirrored how we began our previous report (3). “Traditionally, bacteriostatic antibiotics are agents able to arrest bacterial growth. Despite being traditionally viewed as unable to kill bacterial cells, when they are used clinically, the outcome of these drugs is frequently as effective as when a bactericidal drug is used.” However, we believe that the remainder of our introduction in the current report makes our intent abundantly clear.

We do believe there is another major question of what to do with the words “bacteriostatic” and “bactericidal.” The collective would have you believe that the science is settled, and these words are antiquated relics of bygone science. Should our report have been about *Bacillus coli*, we would agree. The concept of bacteriostatic and bactericidal is still taught in microbiology classrooms (7–9); guidelines published by IDSA still describe drugs as bacteriostatic (10); indeed, not even 2 months ago we had a discussion with a Professor of Infectious Diseases about his hesitancy to treat Staphylococcal bacteremia with linezolid solely due to it being a bacteriostatic drug. These realities raise a question of solvency. We would suggest continuing to publish manuscripts using these words that demonstrate the insufficiency of bacteriostatic and bactericidal to describe the phenomenology underlying these agents. We would suggest publishing perspectives and opinions suggesting more useful language to adopt (11). The collective would seem to argue for banning these words—something that is both impossible and has no utility. We must change the minds of treating physicians, scientists, and our students.
